# Advancing indigent healthcare services through adaptive reuse: repurposing abandoned buildings as medical clinics for disadvantaged populations

**DOI:** 10.1186/s12913-017-2752-8

**Published:** 2017-12-13

**Authors:** James K. Elrod, John L. Fortenberry

**Affiliations:** 1 Willis-Knighton Health System, 2600 Greenwood Road, Shreveport, LA 71103 USA; 2 LSU Shreveport, 1 University Place, Shreveport, LA 71115 USA

**Keywords:** Adaptive reuse, Repurposing, Medically underserved populations, Charity care

## Abstract

**Background:**

Challenges abound for healthcare providers engaged in initiatives directed toward disadvantaged populations, with financial constraints representing one of the most prominent hardships. Society’s less fortunate typically lack the means to pay for healthcare services and even when they are covered by government health insurance programs, reimbursement shortcomings often occur, placing funding burdens on the shoulders of establishments dedicated to serving those of limited means. For such charitably-minded organizations, efficiencies are required on all fronts, including one which involves significant operational costs: the physical space required for care provision.

**Discussion:**

Newly constructed buildings, whether owned or leased, are expensive, consuming a significant percentage of funds that otherwise could be directed toward patient care. Such costs can even prohibit the delivery of services to indigent populations altogether. But through adaptive reuse—the practice of repurposing existing, abandoned buildings, placing them back into service in pursuit of new missions—opportunities exist to economize on this front, allowing healthcare providers to acquire operational space at a discount. In an effort to shore up related knowledge, this article profiles Willis-Knighton Health System’s development of Project NeighborHealth, an indigent clinic network which was significantly bolstered by the economies associated with adaptive reuse.

**Conclusions:**

Despite its potential to bolster healthcare initiatives directed toward the medically underserved by presenting more affordable options for acquiring operational space, adaptive reuse remains relatively obscure, diminishing opportunities for providers to take advantage of its many benefits. By shedding light on this repurposing approach, healthcare providers will have a better understanding of adaptive reuse, enabling them to make use of the practice to improve the depth and breadth of healthcare services available to disadvantaged populations.

## Background

Addressing indigent populations is profoundly challenging, with financial constraints representing one of the most prominent hardships [1, 2]. Society’s disadvantaged typically lack the means to pay for healthcare services and even when they are covered by government health insurance programs, reimbursement shortcomings often occur and deter access to care. This, in turn, places funding burdens on the shoulders of charitably-minded healthcare entities dedicated to serving the less fortunate, often on very limited budgets. Such budgets, of course, require prudent financial decisions to stretch the value of each and every available charitable dollar. Operational costs in particular must be monitored closely and controlled well, ideally permitting the largest share possible of available funds to be directed toward elevating the health status of the needy.

Of all of the costs faced by providers of charitable healthcare services, one of greatest magnitude centers on the physical space required for care provision [3, 4]. Newly constructed buildings, whether owned or leased, are expensive, consuming a significant percentage of funds that otherwise could be directed toward patient care. Associated costs can even be prohibitive, preventing the delivery of services to disadvantaged populations altogether. But through adaptive reuse—the practice of repurposing existing, abandoned buildings, placing them back into service in pursuit of new missions—opportunities exist to economize on this front, allowing healthcare providers to acquire operational space at a discount. Savings here permits a greater portion of scarce resources to be used for the actual delivery of medical care to patients, bolstering the amount of indigent care that can be delivered by a given institution. It even affords opportunities for healthcare providers to enter communities which otherwise would remain unserved [5].

Despite its potential to bolster charity care by presenting more affordable options for acquiring operational space, adaptive reuse remains relatively obscure. While the practice has been portrayed extensively in sectors such as retail, hospitality, and housing [6–8], adaptive reuse historically has received very little attention in the health services literature, with cursory accounts constituting the bulk of efforts (e.g., [9–13]). This is changing thanks to recent comprehensive attention (e.g., [5]), but the body of healthcare-industry specific knowledge associated with adaptive reuse remains sparse, diminishing opportunities for providers to gain an awareness and understanding of the practice.

In an effort to bolster the literature of adaptive reuse specifically as it is applied in healthcare settings, this article profiles Willis-Knighton Health System’s use of the practice as it went about establishing a network of clinics dedicated to serving the indigent. The resulting network, known as Project NeighborHealth, answered prominent calls for the disadvantaged to gain better access to healthcare services. The network has positively impacted the lives of thousands who have no other means of receiving health and medical care, but without the efficiencies afforded by adaptive reuse, its depth and breadth would be significantly reduced. In presenting details describing Willis-Knighton Health System’s actions which led to the realization and ultimate success of Project NeighborHealth, adaptive reuse is profiled comprehensively, affording providers with the critical knowledge necessary to make use of the practice to bolster charitable healthcare initiatives broadly.

### Foundations of adaptive reuse

Repurposing has become a common buzzword in modern times, holding a place in the language of the general public resulting from its use on many fronts. Popular television shows focus on searching for discarded items which have exhausted their first lives, placing the discoveries back into circulation often as artifacts of nostalgia serving a new and different purpose than that originally intended. Home improvement shows frequently present accounts where rooms designed for a particular use are repurposed as new and different spaces (e.g., converting a garage into a guest bedroom). Further, growing concerns for the environment have led to increasing calls for consumers to reuse myriad products, often in creative manners, essentially repurposing the items to reduce the burden that they otherwise would place on landfills. The same general repurposing approach demonstrated in these popular portrayals—identifying items that are exhausted or otherwise not realizing their full potential and taking action steps to redirect them to useful service—is also used in commercial real estate to generate new lives for abandoned, unproductive properties. The approach, termed adaptive reuse, offers pathways for acquiring space at a significant discount to that of new construction, all while generating numerous benefits associated with the restoration of operations at once idled sites [14–17]. Formally defined, adaptive reuse is “the practice of identifying, acquiring, renovating, and placing back into service a building or similar structure for a purpose different than that for which it was originally designed” [5], p. 6.

Abandoned buildings, the core requirement of adaptive reuse projects, exist in many communities, arising due to a variety of circumstances ranging from successes which prompt relocations to failures which shutter establishments. These idled properties, even if in good repair, often are not enticing candidates to prospective tenants given inactivity and the usual stark appearance of closed establishments, typically padlocked, boarded up, and powered down [5]. But those familiar with adaptive reuse look beyond the superficial to see potential even in the grimmest of circumstances. This permits them to leverage buyer disinterest to realize acquisitions at significant discounts. Such efficiencies immediately increase the prospects for conducting viable operations at the resulting establishment, given the reduced tow of associated real estate expenditures. This is especially critical for healthcare providers engaged in initiatives directed toward indigent populations, as these services usually are delivered for reduced reimbursement rates and, very often, no reimbursement at all. Clearly in such cases, economization must occur at every available opportunity. Adaptive reuse greatly facilitates operational frugality and, as Willis-Knighton Health System experienced, it represents an excellent pathway for efficiently bolstering healthcare services directed toward the underserved [5, 18].

### Willis-Knighton Health System, charitable care, and adaptive reuse

Headquartered in Shreveport, Louisiana, Willis-Knighton Health System is a nongovernmental, not-for-profit healthcare provider delivering comprehensive health and wellness services through multiple hospitals, numerous general and specialty medical clinics, an all-inclusive retirement community, and more. The system holds market leadership in its served region, centered in the heart of an area known as the Ark-La-Tex, where the states of Arkansas, Louisiana, and Texas converge. Willis-Knighton Health System’s origins date to 1924 with the establishment of Tri-State Sanitarium, founded to address the healthcare needs of the burgeoning population of west Shreveport. Sold in 1929 to Drs. James Willis and Joseph Knighton, the establishment continued operations and, in 1952, it was renamed in honor of Drs. Willis and Knighton. Today, resulting from decades of planning and development, Willis-Knighton Health System possesses an enduring legacy of excellence, robustly addressing the health and wellness needs of the citizenry, notably including those without the means to pay for healthcare services.

While Shreveport and surrounding municipalities clearly show all of the signs of a modern, progressive marketplace, many residents have been left behind, struggling daily to acquire food, shelter, healthcare, and other necessities of life. According to the United Way’s ALICE (Asset Limited, Income Constrained, Employed) Report which monitors those who are employed yet still unable to afford basic necessities and those suffering from poverty, 45% (114,912) of the households in northwest Louisiana are struggling to make ends meet. Of these households, 23% are ALICE and 22% are poverty stricken, making the region one of the poorest in the nation [19]. To help address this alarming situation, Willis-Knighton Health System has invested heavily in directives which improve opportunities for the poor. Today, the system delivers more charitable care than all other healthcare providers in the marketplace combined. Without Willis-Knighton Health System’s efforts to address the region’s most vulnerable populations, the state of health and wellness would be dire. In fact, some disadvantaged neighborhoods would be entirely without healthcare services. With care recipients being uninsured or underinsured, however, frugality is absolutely essential for any hope of serving the poor successfully. This compelled Willis-Knighton Health System to turn to adaptive reuse to capitalize on the practice’s economies.

Willis-Knighton Health System’s experience with adaptive reuse dates back to the 1970s and came about by necessity. Executives desired to expand the establishment’s footprint beyond its historic served market of west Shreveport, but funding for achieving growth ambitions was scarce. This necessitated highly efficient practices which stretched the value of the dollar, permitting the accomplishment of more with less. Executives thought creatively on options for expanding services economically and the notion of repurposing abandoned buildings to serve medical missions came to light. Early adaptive reuse endeavors proved the efficiency and effectiveness of the approach, confirming earlier assumptions and warranting further use. To date, Willis-Knighton Health System has completed over 20 adaptive reuse projects, with more currently underway [5]. Through Willis-Knighton Health System’s many adaptive reuse projects, a significant pool of expertise was acquired, giving executives extensive insights into the practice. In an earlier article profiling the system’s various adaptive reuse experiences, opportunities associated with adaptive reuse were noted as follows.

1. Financial savings, courtesy of the highly-favorable pricing often tied to abandoned properties and the potential to use all or part of the existing infrastructure in renovations, reducing building materials and labor costs;
2. Access to premium locations, as adaptive reuse effectively puts back into play sites that already are occupied by existing structures, many of which have outstanding visibility and accessibility;
3. Community renewal, as adaptive reuse efforts place abandoned and often blighted properties back into productive service, improving the scenic beauty and vitality of given communities; and
4. Resource conservation, as repurposed abandoned buildings escape demolition and the burdens that it places on landfills and they also carry fewer natural resource requirements than new construction projects due to recycled infrastructure [5].

In the same article, obstacles associated with adaptive reuse were communicated, with these including the following.

1. Insufficient or nonexistent availabilities, in that sometimes even desires to pursue adaptive reuse cannot be accommodated because a suitable candidate property is not available;
2. Excessive renovation costs, as some adaptive reuse candidates require renovations of such magnitude that their pursuit cannot be justified financially;
3. Disagreements with stakeholders over the property’s intended reuse, whereby the new mission of the proposed establishment is not welcomed by community stakeholders; and
4. Zoning difficulties, in cases where the required permissions from regulatory bodies cannot be acquired by the proposed establishment [5].

Given differences between and among candidate properties, each adaptive reuse case is situation dependent. While obstacles certainly can appear, many can be traversed successfully and some projects will be completely free of barriers. Being that most of the opportunities afforded by adaptive reuse are unavailable through any other method of spatial expansion, exploration of candidates indeed is worthwhile. After numerous successes overcoming associated obstacles and capitalizing on opportunities, adaptive reuse has earned a firm place in Willis-Knighton Health System’s operational culture and it is the first consideration whenever executives are faced with a spatial acquisition need that cannot be accommodated by renovating an existing property. Only after adaptive reuse opportunities have been explored and deemed to be either unavailable or unviable are new construction pathways pursued. The reservoir of adaptive reuse skills and abilities acquired by Willis-Knighton Health System over many years proved especially helpful in establishing its indigent clinic network, Project NeighborHealth.

### Willis-Knighton Health System's Project NeighborHealth

Established in 1995, Project NeighborHealth is a Willis-Knighton Health System initiative designed, developed, and operated to serve disadvantaged populations in Shreveport and the greater region. Beginning as a single clinic, Project NeighborHealth now is comprised of 11 medical establishments, placed intentionally inside or in close proximity to neighborhoods of extreme poverty, facilitating access to care and fostering medical compliance. Specifically, eight of Project NeighborHealth’s clinics are located within underprivileged neighborhoods, two are located in close proximity to challenged communities, and one unit—a mobile clinic based near a disadvantaged area—ventures into underserved communities throughout the region specifically to administer vaccinations to underprivileged children. Collectively, Project NeighborHealth serves as a critical healthcare lifeline for the less fortunate in the marketplace.

Willis-Knighton Health System’s interest in serving indigent populations has been historic, but formal efforts to establish clinics which were strategically located in or near poverty-stricken communities did not begin until the 1990s, occurring in tandem with Willis-Knighton Health System’s dramatic expansion resulting in market leadership. The system’s increased footprint and significant market share afforded financial resources which permitted funding of a wealth of pursuits, notably including efforts to shore up access gaps in the marketplace. On researching critical voids in community health, executives identified a number of disadvantaged neighborhoods in the vicinity whose residents essentially were shut off from accessing healthcare services. Residents in these areas did not have sufficient access to personal transportation and public transportation was either limited or unavailable in their neighborhoods, reducing possibilities for them to travel to regional medical providers. In some cases, the neighborhoods were located in remote areas, making travel for receipt of medical services a significant hardship, even with access to transportation. In all cases, healthcare services in these neighborhoods were either woefully inadequate or completely nonexistent, with the end result being isolated populations with myriad unmet healthcare needs [18].

On further investigation, Willis-Knighton Health System discovered exceptionally high death rates in these communities, with common culprits being heart disease, diabetes, and similar illnesses which potentially could have been prevented with sufficient access to healthcare services. Leaders in these neighborhoods further conveyed to executives the access hardships faced by residents and the associated consequences on their lives and encouraged Willis-Knighton Health System’s assistance [18]. With verification of gaps in community health, calls by stakeholders to help resolve issues, and the ability to fund associated interventions, Willis-Knighton Health System decided to establish an indigent clinic network as a means of bolstering health and wellness opportunities for the region’s most vulnerable. Given that services would be offered with no expectation of payment, a premium was placed on achieving efficiencies, something which encouraged adaptive reuse pursuits at every possible opportunity.

### Project NeighborHealth and adaptive reuse

**Fig. 1:**
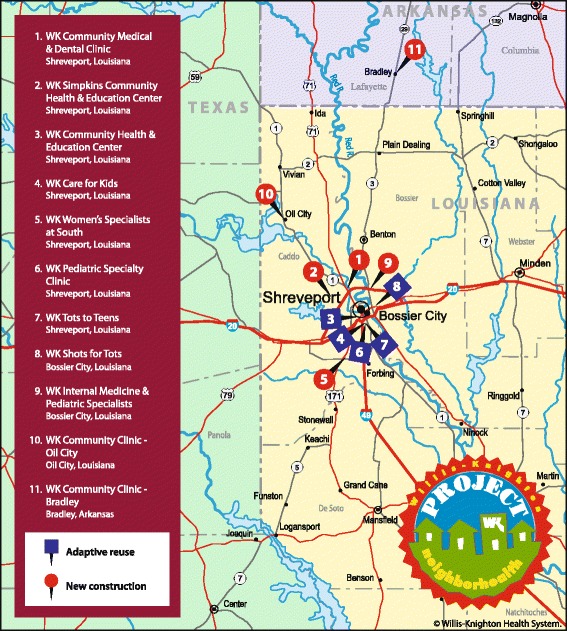
A map presenting Willis-Knighton Health System’s Project NeighborHealth

Willis-Knighton Health System approached the development of Project NeighborHealth with the same philosophy used in its development of prior expansion projects: If the given expansion cannot be addressed by renovating existing space, adaptive reuse options are first explored. If found to be nonexistent or unviable, new construction options are then pursued. In developing Project NeighborHealth, in some cases, acceptable adaptive reuse candidates were not available, leading to new construction pursuits. In other cases, attractive adaptive reuse candidates were identified and pursued. A map presenting each clinic’s location and origin as either an adaptive reuse or new construction project is supplied in Fig. 1. As identified in the figure, five of Project NeighborHealth’s 11 clinics emerged by repurposing abandoned buildings which were comprehensively revitalized as like-new, state-of-the-art facilities, generating savings which would not have been possible via equivalent new construction, reducing the financial burden associated with delivering charity care. This savings allowed Willis-Knighton Health System to direct a greater share of each dedicated charitable healthcare dollar toward care provision and also permitted the realization of clinics which otherwise would have been cost prohibitive to establish. All told, adaptive reuse significantly magnified the depth and breadth of Project NeighborHealth, enhancing its resulting impact on indigent populations in the Ark-La-Tex region of the United States.

### Project NeighborHealth's WK Community Health and Education Center

**Fig. 2:**
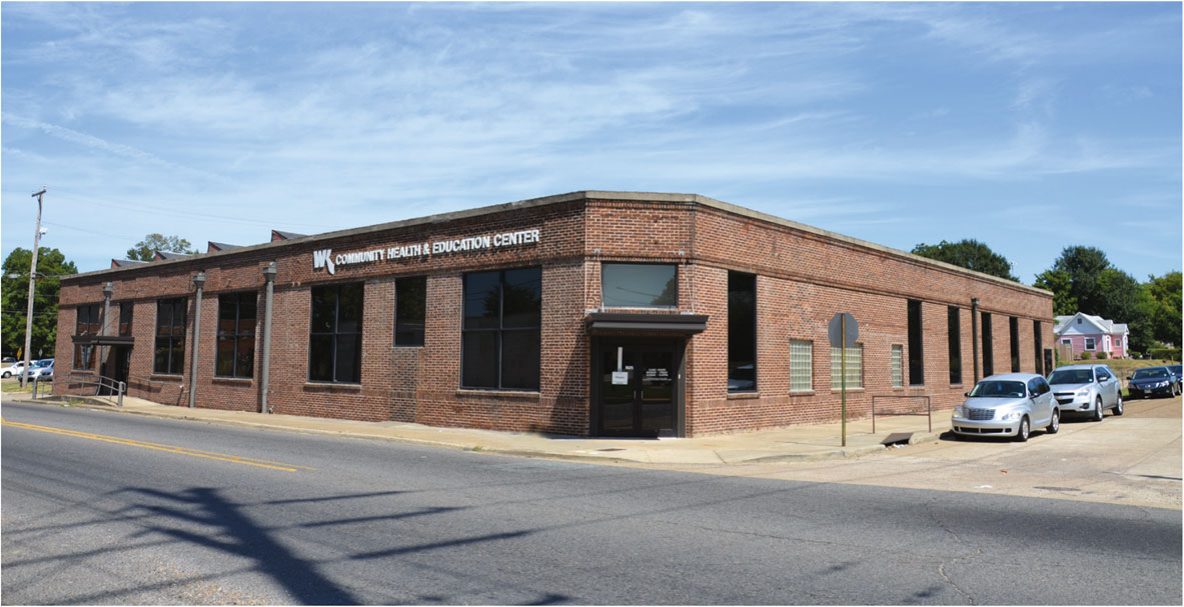
Project NeighborHealth’s WK Community Health and Education Center

**Fig. 3:**
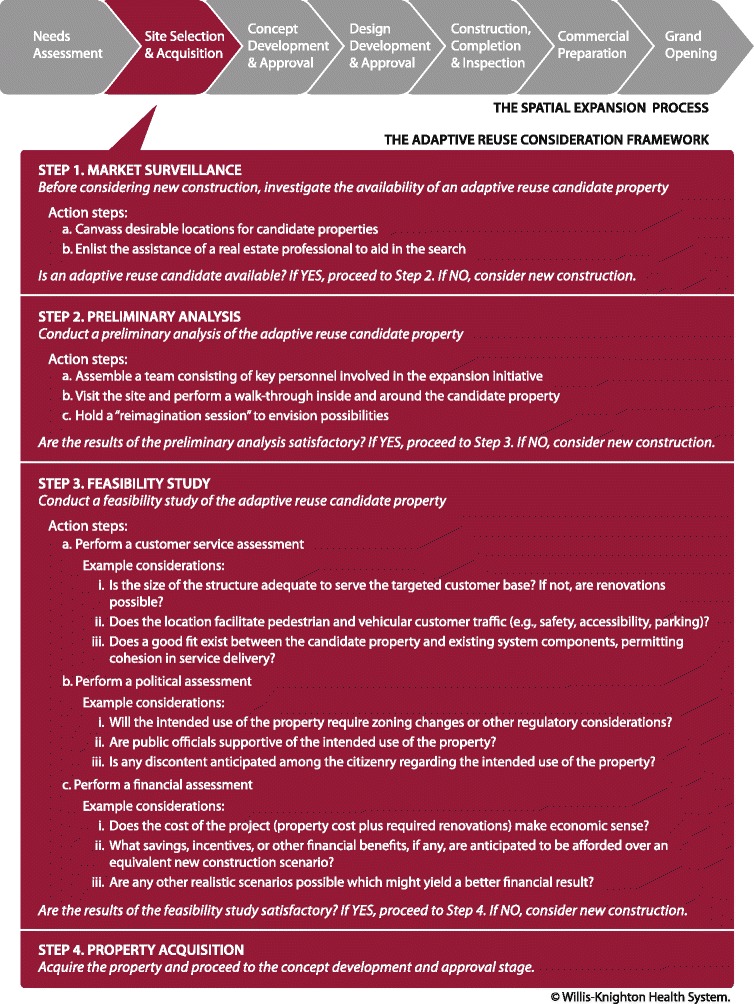
A protocol for considering adaptive reuse

To shed further light on adaptive reuse and its implementation, a detailed profile of an actual adaptive reuse endeavor which led to an operational Project NeighborHealth clinic will be helpful. Of all of Project NeighborHealth’s clinics, the WK Community Health and Education Center, located on Pierre Avenue in Shreveport’s Allendale neighborhood and presented photographically in Fig. 2, is perhaps the most intriguing from an adaptive reuse standpoint. In evaluating adaptive reuse projects, Willis-Knighton Health System follows a standard protocol, as shown in Fig. 3, which it developed over its various associated experiences. As portrayed in the figure, situated within the site selection and acquisition stage of the usual and customary process associated with realizing a spatial expansion, opportunities exist to consider adaptive reuse candidates following a 4-step protocol, termed the Adaptive Reuse Consideration Framework. The WK Community Health and Education Center project followed this protocol, but entailed some interesting variations, courtesy of substantial dedication, commitment, and involvement on the part of neighborhood stakeholders. The steps, along with insights pertaining to the establishment of this particular Project NeighborHealth clinic, are described as follows.

#### Step 1: market surveillance

The Adaptive Reuse Consideration Framework begins with executives exploring the general location where an expansion is needed to determine if an abandoned and available building that can accommodate the desired application exists, something ascertained by personal site visits and often facilitated with the use of real estate professionals. If a viable candidate property cannot be identified, new construction is pursued, but if an adaptive reuse opportunity is present, the evaluation proceeds to the next step in the framework [5]. In the case of what would become Project NeighborHealth’s WK Community Health and Education Center, the usual burdens associated with this particular step were significantly reduced, thanks to the vision and generosity of Dr. E. Edward Jones, Sr. (1931–2016), pastor of Galilee Baptist Church. Dr. Jones had observed the success associated with Willis-Knighton Health System’s initial Project NeighborHealth clinic, the WK Simpkins Community Health and Education Center. Located in north Shreveport and established in 1995, this particular clinic had positively impacted health and wellness in its neighborhood of operation and Dr. Jones was desirous of seeing the same occur in Shreveport’s Allendale neighborhood, home of his ministry and congregation at Galilee Baptist Church.

Galilee Baptist Church owned an adjacent, abandoned building formerly occupied by a commercial laundry and dry cleaning company which had ceased business operations years earlier. Constructed in the early twentieth century, this approximately 15,000 square foot building was sitting idle and it occurred to Dr. Jones that it presented an opportunity for filling healthcare access gaps which existed in the Allendale neighborhood. Knowing of Willis-Knighton Health System’s commitment to addressing the healthcare wants and needs of the disadvantaged, Dr. Jones reached out to executives, proposed the establishment of a Project NeighborHealth clinic in the community, and offered to provide the idled property at no charge in exchange for the system repurposing the building and grounds and operating the resulting medical clinic. As Willis-Knighton Health System was actively developing Project NeighborHealth, this gracious gesture was perfectly timed. With an adaptive reuse candidate building being offered at no cost thanks to the generosity of Dr. Jones and Galilee Baptist Church, Willis-Knighton Health System proceeded to conduct a preliminary analysis, the next step of the Adaptive Reuse Consideration Framework.

#### Step 2: preliminary analysis

The preliminary analysis step of the Adaptive Reuse Consideration Framework involves conducting an onsite inspection of the candidate property. Here, an evaluation team consisting of executives, engineering and construction personnel, architecture and design consultants, and other key staff members view firsthand the current state of the premises and determine its potential to be repurposed as the envisioned establishment [5]. Via site investigations, Willis-Knighton Health System’s evaluation team found the building offered by Galilee Baptist Church to be a highly suitable, especially solid structure. Related to its period of design, the building featured an intriguing architectural feature—a saw-tooth roof permitting natural light to enter the facility—which the evaluation team intended to preserve, should the project move forward. The layout of the building was not well suited for the delivery of healthcare services, with a moderate level of demolition of interior structures being required to effect a proper layout, but this is often the case in most any comprehensive repurposing project. Electrical and mechanical infrastructure, including environmental systems, needed upgrades to meet modern standards and suit the consumer-focused application of healthcare delivery which differed considerably from the building’s original industrial application involving commercial laundry activities.

After a comprehensive site assessment, Willis-Knighton Health System’s evaluation team viewed the building to be an excellent adaptive reuse candidate which, through modern renovation techniques and proper investment, could emerge as a like-new establishment designed and equipped to successfully deliver health and wellness services to those residing in and around the Allendale neighborhood. Through ensuing reimagination sessions, the evaluation team and members of Galilee Baptist Church arrived at a working concept which entailed establishing a comprehensive health and wellness facility that included a medical clinic, wellness center featuring the latest fitness equipment, and a computer laboratory for children in the neighborhood. The candidate building clearly had enough capacity to feature this array of services.

#### Step 3: feasibility study

Per the Adaptive Reuse Consideration Framework, following a successful preliminary analysis, a feasibility study is conducted to more deeply examine the adaptive reuse candidate. Specifically, the building’s potential on customer service, political, and financial fronts is examined. The customer service assessment centers on evaluating the candidate property’s capacity to successfully address the needs of those who will patronize the proposed entity. The political assessment focuses on determining whether zoning and other regulations will permit the establishment of the envisioned facility. Further, stakeholder support for the operation is assessed to determine the political feasibility of the concept. The financial assessment examines the costs associated with repurposing the candidate property in an effort to determine if the pursuit offers a more compelling financial case than that of new construction or any available alternative scenario [5].

With an extensive internal, technical knowledge base associated with healthcare facility building and construction, coupled with the assistance of partner architecture and engineering firms, Willis-Knighton Health System’s team drew up formal plans for the proposed Project NeighborHealth clinic, refining the design over several planning sessions. The resulting design met aesthetics, ergonomics, access, and related criteria required for delivering the targeted services. Further, transit pathways to resources within the system were envisioned and determined to be adequate for providing necessary support services for the clinic, giving executives confidence regarding the design and the property’s ability to meet the needs of patients. Politically, the project had tremendous community support from private, public, and not-for-profit parties, given that the idled building would be put back into use for the purpose of supplying vitally-needed healthcare services. Further, zoning and related regulatory matters were determined to be accommodating to the revised use of the property, clearing associated political hurdles with ease. Financially, the project had a significant head start in that the building acquisition carried no associated costs, so Willis-Knighton Health System’s executives concentrated on the renovation costs required to realize the envisioned clinic and the costs of operation going forward. Courtesy of the work of commissioned architects and engineers, formal cost estimates were in hand. As it was well understood that the proposed clinic would provide services with no expectation of remuneration, attention was directed toward determining whether anticipated disbursements forwarded annually through Willis-Knighton Health System’s charitable efforts could cover the costs associated with the envisioned indigent clinic. The system’s robust success and resulting financial resources, together with projections indicating favorable conditions going forward, gave executives confidence that the proposed clinic could be supported via its charitable arm. Essentially, by redistributing resources, directing a portion from prosperous service lines into indigent care, the proposed clinic, through such surrogacy, could be supported. Ultimately, through a very unique scenario which included a donated building provided by Galilee Baptist Church and significant investment on the part of Willis-Knighton Health System, the project was deemed to be worthy of pursuit as a charitable endeavor.

#### Step 4: property acquisition

Property acquisition, the final step in the Adaptive Reuse Consideration Framework, occurs if the candidate property successfully passes the assessments set forth in prior steps. If so, the project advances to the concept development and approval stage, eventually leading to a repurposed, like-new establishment which meets the expansion needs of the institution and once again delivers value in the community [5]. With the candidate building deemed to be viable to support the mission of the envisioned clinic, Willis-Knighton Health System agreed to pursue the project. Rather than a direct purchase, the property was acquired via an agreement between Willis-Knighton Health System and Galilee Baptist Church meeting the terms noted earlier. In forthcoming months, Willis-Knighton Health System developed the site fully. It reemerged in 1998 as Project NeighborHealth’s WK Community Health and Education Center, carrying a total cost of approximately $1.4 million. Had Willis-Knighton Health System opted to pursue new construction, the figure would have been approximately $150,000 higher. Due to the extent of renovations required to bring the building to proper operational standards, the savings here was somewhat modest compared to some of Willis-Knighton Health System’s other adaptive reuse projects, but still reflected a healthy sum for direction toward other mission-critical activities. Annually, the clinic now delivers care to approximately 3500 patients in the Allendale neighborhood. It remains the only source of healthcare for many area residents.

## Conclusions

With knowledge of adaptive reuse and its operation, it is fairly easy to understand its potential for bolstering healthcare services directed toward the indigent. By acquiring a suitable, abandoned building and repurposing it for less than the cost of new construction, opportunities to enhance and expand the depth and breadth of charitable care initiatives emerge. Outreach efforts into medically underserved communities can be effected at a discount to that of new construction, permitting healthcare institutions to enter these markets more economically than they could via other routes. Further, given the economies of adaptive reuse and the practice’s ability to reduce associated real estate expenditures, a greater percentage of each dollar dedicated to charity care can be directed toward addressing the health and wellness needs of patients in these disadvantaged communities.

Willis-Knighton Health System can confirm that without the efficiencies afforded by adaptive reuse, its Project NeighborHealth indigent clinic network would be a shadow of itself. Not only would the number of locations be reduced, but also the depth and breadth of healthcare services provided at each location would be diminished. Essentially, adaptive reuse has allowed Willis-Knighton Health System to accomplish more with less, something welcomed in most any situation, but vital in the delivery of charity care where the costs associated with delivering services largely or completely are funded by the care provider. Notably, the practice has afforded a wealth of secondary benefits associated with replacing idled properties with active operations which deliver value on multiple levels in their given neighborhoods.

Significantly, adaptive reuse is one of the most accessible of approaches, with virtually any healthcare provider desirous of making use of the practice being able to do so. The primary operational requirement is simply the knowledge of the approach and the willingness to consider repurposing opportunities whenever spatial expansion needs emerge. This, coupled with the implementation guidance supplied by Willis-Knighton Health System’s Adaptive Reuse Consideration Framework, can bring the practice clearly within the grasp of most any healthcare provider, affording a helpful option that can magnify the existence and impact of health and medical initiatives directed toward disadvantaged populations.
